# Processing of Angelica (*Angelica sylvestris* L.) into a functional jam with addition of carob and cinnamon extracts: Evaluation of sensorial, physicochemical, and nutritional characteristics and in vitro bioaccessibility of phenolics

**DOI:** 10.1002/fsn3.4476

**Published:** 2024-10-07

**Authors:** Elif Koç Alibaşoğlu, Büşra Acoğlu Çelik, Fatma Duygu Ceylan, Özüm Özoğlu, Ertürk Bekar, Esra Çapanoğlu, Canan Ece Tamer, Mihriban Korukluoğlu, Ömer Utku Çopur, Perihan Yolci Ömeroğlu

**Affiliations:** ^1^ Food Engineering Department, Faculty of Agriculture Bursa Uludag University Bursa Turkey; ^2^ Science and Technology Application and Research Center (BITUAM) Bursa Uludag University Bursa Turkey; ^3^ Food Engineering Department, Faculty of Chemical and Metallurgical Engineering Istanbul Technical University Istanbul Turkey

**Keywords:** Angelica jam, *Angelica slyvestris*, bioactive compound, traditional food

## Abstract

A traditional Angelica jam has been produced from stems of *Angelica sylvestris* L. growing at the foot of Mount Uludag in Bursa for many years. The aim of this study was to develop new functional formulations of Angelica jam by adding carob and cinnamon extracts, and to evaluate the effect of jam processing and the extracts' addition on the sensory attributes, physicochemical and nutritional characteristics of Angelica jams as well as in vitro bioavailability of phenolics. The main physicochemical properties of the jams including total water‐soluble dry matter (Brix value), pH, invert sugar content, total dietary fiber content, and 5‐(hydroxymethyl) furfural (HMF) ranged between 71.80–72.70 *°Bx*, 3.65–3.91, 39.30%–49.60%, 0.70–0.90 g/100 g, and 91.00–105.50 mg/kg, respectively. The most abundant minerals were potassium (K), calcium (Ca), magnesium (Mg), phosphorus (P), and iron (Fe) within a concentration range of 80.49–388.67 mg/kg, 111.10–135.00 mg/kg, 24.00–43.28 mg/kg, 7.57–28.06 mg/kg, and 0.52–1.14 mg/kg, respectively. Sensory analysis revealed that general acceptability of Angelica jams in a 9‐point hedonic scale was above 7. The highest antioxidant capacity values of Angelica jam and its functional forms fortified with carob and cinnamon extracts were obtained as 96.68 ± 11.30 mg TE/100 g DM, 248.49 ± 17.78 mg TE/100 g DM, and 193.11 ± 16.06 mg TE/100 g DM, respectively, with the ABTS (2,2′‐azino‐bis‐3‐ethylbenzothiazoline‐6‐sulfonic acid) method. It was observed that the main phenolic compound of the jams was caffeic acid. Depending on the type of extract added into the formulations, trans‐cinnamic acid and gallic acid (GA) became predominant phenolics. Total antioxidant capacity (TAC) of *Angelica sylvestris* stem decreased by 72%–85% with the heat treatment applied during jam processing. While total phenolic content (TPC) and total antioxidant capacity (measured by the DPPH (1,1‐diphenyl‐2‐picrylhydrazyl) method) increased as a result of intestinal digestion of the jam, it was observed that bioaccessibility of most of the phenolic compounds decreased after intestinal digestion of the jams.

## Graphical abstract

The aim of this study was to develop new functional formulations of Angelica jam by adding carob and cinnamon extracts, and to evaluate the effect of jam processing and the extracts' addition on the sensory attributes, physicochemical and nutritional characteristics of Angelica jams as well as in vitro bioavailability of phenolics. Addition of both extracts to Angelica jam improved physicochemical and bioactive properties of the final product. Therefore, future studies can include the investigation of different types of aromatic and medical plant extracts that can be incorporated into traditional jam formulations to enhance their functional properties and increase product diversity.

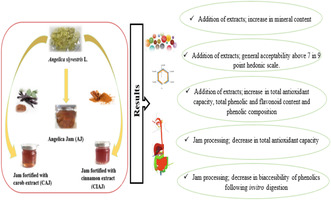

## 1 INTRODUCTION

The genus *Angelica* L. with its 80 species is widely spread in the countries where the northern temperature zone exists (Özek et al., 2008). *Angelica sylvestris* L., one of the two species of the genus *Angelica* L. common in Turkey, can be found at the foot of Mount Uludag in Bursa, which boasts a flora containing many endemic plants (Daşkın & Kaynak, 2012; Stanković et al., 2016). The flowers of *A. sylvestris* L. are in open form, and multilayered umbrella shape, and its leaves are greenish and powdery pink close to white (Stpiczyńska et al., 2015). The studies reported in the literature revealed that *A. sylvestris* L. shows antioxidant capacity with its bioactive compounds, including coumarins, sterols, phenolic acids, and limonene (Ağalar et al., 2020; Karaçelik et al., 2022; Murphy et al., 2004; Özek et al., 2008; Porrello et al., 2023; Raal et al., 2022; Saleh et al., 2023; Stpiczyńska et al., 2015; Tykheev et al., 2020). Moreover, it exhibits antimicrobial activity proven against some selected bacterial and fungal strains (Canli et al., 2016; Dede et al., 2023). In this context, it was reported that *A. sylvestris* L. was used in traditional medicine for the treatment of digestive and respiratory system diseases, as a nutritional supplement for women, and as an ingredient for the local remedy called Mesir paste during the Ottoman period (Akgun et al., 2022; Daşkın & Kaynak, 2012; Stanković et al., 2016). Moreover, a traditional jam has been produced from stems of *A. sylvestris* L. growing at the foot of Mount Uludag in Bursa for many years (Daşkın & Kaynak, 2012; Koç & Yolcı Ömeroğlu, 2019).

Jam can be prepared by boiling half or smaller pieces of fruit and vegetables with sucrose, pectin, organic acid, and other ingredients and brought to a certain consistency (Anonymous, 2006; Koç & Yolcı Ömeroğlu, 2019). In jam production, there are many operations including cutting, shredding, and heat treatment that can affect the polyphenol content of the product. These processes not only allow the release of bioactive compounds from the matrix but also provide higher bioavailability through the digestion systems, by providing a reduction in particle size and increased surface area. However, these processes can also cause a negative effect by degradation and/or oxidation of cell integrity and the reaction of polyphenols with enzymes such as polyphenol oxidase that causes a decrease in the total phenolic content (TPC) of the final product (Belović et al., 2017; Kamiloglu et al., 2015; Shinwari & Rao, 2018). In this context, Koç and Yolcı Ömeroğlu (2020) evaluated the functional properties of Angelica jam, by revealing its total antioxidant capacity (TAC), total phenolic and total flavonoid contents by assessing in vitro bioaccessibility of those components. It was concluded that the functionality and product variety of the jam should be increased by optimization of the formulation.

Consumer's desire to have healthy products without changing their traditional eating habits has brought the functionality of traditional foods to the agenda. For this purpose, functional foods are obtained by fortification or enrichment of phenolic substances, antioxidants, nutritional fibers, oligosaccharides, vitamins, plant sterols, prebiotics, and probiotics (Dayısoylu et al., 2014). In recent years, extracts of aromatic and medicinal plants such as carob and cinnamon have attracted interest due to their strong antioxidant capacity (Chait et al., 2020; Dvorackova et al., 2015; Kim et al., 2014; Loullis & Pinakoulaki, 2018; Ostroschi et al., 2018). Consequently, they have been incorporated into formulations to enhance the functionality of various foods (Ibrahim et al., 2020; Ostroschi et al., 2018; Ribes et al., 2018; Yin et al., 2019). There are limited studies on increasing the functional properties of the jam consumed frequently at breakfast (Sayuti et al., 2015; Loizzo et al., 2021). However, to the best of our knowledge, this study is the first reported in the literature to utilize cinnamon and carob extracts to produce functional jam. Considering the information and needs reported in the literature, this study aimed to develop new functional formulations of Angelica jam by adding carob and cinnamon extracts, and to evaluate the effect of jam processing and extracts addition on the sensory attributes, physicochemical and nutritional characteristics of Angelica jams as well as in vitro bioavailability of phenolics. Additionally, the main ingredient of the jam, *A. sylvestris* L. stem harvested from Mount Uludag, was characterized to reveal its physicochemical, antifungal, and bioactive properties.

## 2 MATERIALS AND METHODS

### 2.1 Chemicals and reagents

For sugar composition analyses, neat standards of glucose, fructose, sucrose, and maltose were purchased from Dr. Ehrenstorfer with a purity higher than 98.5%. A neat standard of 5‐(hydroxymethyl) furfural was provided by Merck with a purity of <97%. For the simulation of in vitro gastrointestinal (GI) digestion system, dialysis bags (Membra‐Cel MD34‐14 × 100 CLR) from Serva Electrophoresis GmbH (Heidelberg, Germany) were purchased. Caffeic acid, syringic acid, rosmarinic acid, trans‐cinnamic acid, gallic acid, rutin, and epicatechin with a purity of ⩾98% were used for the quantification of phenolic compounds. Multielement aqueous solution at 1000 mg/L in 2% (wt/wt) nitric acid (HNO_3_) was obtained from Merck. Nitric acid 65% was purchased from Merck (Darmstadt, Germany) with a Suprapur quality. All other chemicals and reagents used for physicochemical composition and phenolic compounds analyses were of analytical or high‐performance liquid chromatography (HPLC) grade and obtained from Sigma‐Aldrich Chemie GmbH & Co. KG (Steinheim, Germany), unless otherwise specified. High‐purity deionized water was prepared using a Milli‐Q purification system (Millipore, France).

### 2.2 Materials for jam processing

*A. sylvestris* L. (AS) was handharvested by contract farmers from the foothills of Uludağ in Bursa, Turkey during the season of May–June 2018. Following the separation of the root and flowers of the plant, they were transported to the laboratory in polyurethane bags within 1–2 days.

The carob and cinnamon extracts were obtained from a local manufacturer in Turkey (Aromsa, Kocaeli). Based on the product specifications, “Carob extract” (FM005837, Aromsa) was extracted from the carob with water and isopropyl alcohol. The carob extract in dark brown color had a slightly viscous liquid appearance and a characteristic carob smell and taste, and its Brix value was measured as 80 *°Bx*. “Cinnamon extract” (FM006975, Aromsa) was extracted from cinnamon powder (*Cinnamomum cassia* L.) with isopropyl alcohol, water, and propylene glycol (E1520). The cinnamon extract was liquid in appearance, dark reddish brown in color, and had a characteristic cinnamon smell and taste, with a Brix value of 70 *°Bx*. Extracts and the plant were kept at 4°C until further analyses.

Sugar, citric acid in crystalline form, and glass jars of 210 mL were purchased from a local market.

### 2.3 Jam processing

**FIGURE 1:**
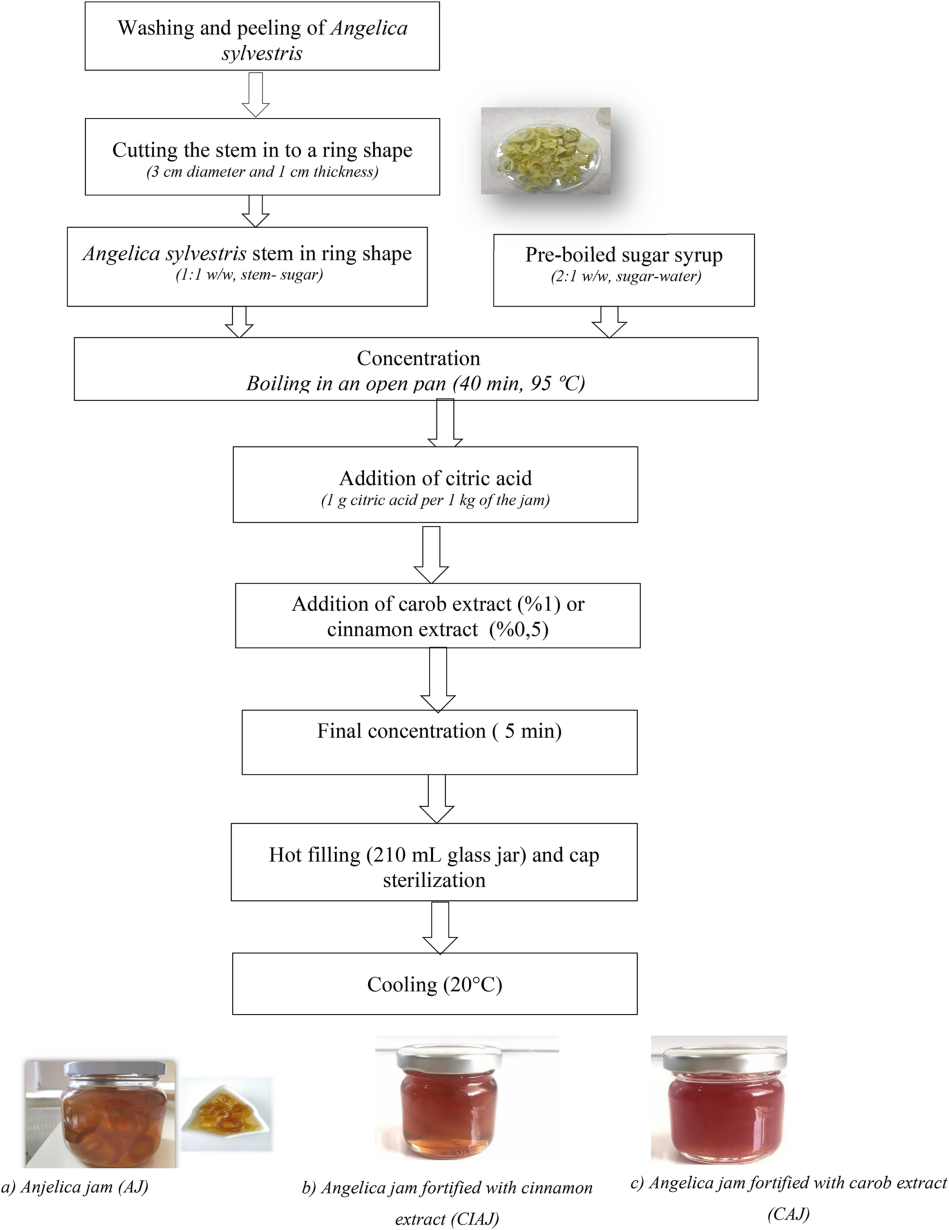
Flowchart for the production of functional formulations of Anjelica jams.

A local producer (Ulus Patisserie) in Bursa, Turkey has been producing Angelica jam in a boutique manner since 1928. The traditional form of Angelica jam was produced based on the local producer's formula (Koç & Yolcı Ömeroğlu, 2019, 2020). The outer peel of the *A. sylvestris* (AS) was separated and the inner stem (*A. sylvestris* stem (ASS)), which was the raw material of the Angelica jam (AJ) was cut into a ring shape with approximately 30 mm in diameter and 10 mm in thickness. Sugar and water in the ratio of 2:1 (w/w) were boiled in an open pan to obtain syrup. Meanwhile, ASS in the same amount of sugar by weight was added into the syrup while stirring constantly. The mixtures were allowed to boil for 40 minutes to reach the desired consistency, followed by the addition of citric acid (approximately 1 g citric acid per 1 kg of the jam). For the production of functional formulations of Angelica jam, cinnamon (Angelica jam fortified with cinnamon extract (CIAJ)) and carob extracts (Angelica jam fortified with carob extract (CAJ)) in 0.5% and 1% ratio were added at that stage to the mixture (Figure 1). Afterward, the heating process was finalized in 5 minutes. The hot products were filled into the 210 mL glass jars, the lid was closed, and they were turned upside down and then left to cool. Jams were stored at 4°C until further analyses (Figure 1). For all analytical studies except those performed for sensory characterization, jams and ASS were ground to a fine powder in liquid nitrogen using a precooled grinder (IKA A11 Basic, IKAWerke GmbH & Co., Germany) and stored at −80°C until further analysis.

### 2.4 Antifungal activity

Yeasts *Saccharomyces cerevisiae* (ATCC 9763) and *Schizosaccharomyces pombe* (Bursa Uludag University Department of Food Engineering) and the mold *Aspergillus niger* ATCC 16404 (*Aspergillus brasiliensis*) were selected as test microorganisms. Yeasts taken from the stock culture (−80°C) were inoculated into tubes containing Malt Extract Broth (Merck) and incubated at 30°C for 24 h. This process was repeated twice to activate the cultures. Active cultures were transferred to Petri dishes containing Malt Extract Agar according to the spreading method and incubated at 30°C for 48 h. Colonies were counted to determine the initial load at the end of incubation. *A. niger* (pure culture) was developed by incubating on flat Malt Extract Agar at 30°C for 7 days. Then, it was mixed with Tween 20 (1%) and its spores were collected followed by storage at 4°C until further analyses. Spore concentration was determined based on the spreading method by transferring spores to the Petri dishes containing Malt Extract Agar followed by 3–7 days of incubation at 30°C (Babu et al., 2003; Korukluoğlu et al., 2006).

*Angelica sylvestris* stem (ASS) was extracted with ethanol (Sigma‐Aldrich) to observe the antifungal activity. The maceration extraction method described by previous researchers (Babu et al., 2003; Canli et al., 2016) was modified as follows. Samples were extracted in ethanol (1:10) with shaking at 100 rpm (revolutions per minute) at room temperature for 3 days. Subsequently, the extract was filtered through filter paper (Whatman No. 1) followed by evaporation of the solvent by a rotary evaporator (Heidolph Laborota 4000, Germany). The residues at the end of evaporation were collected and 100 mg/mL extracts were prepared.

The diffusion method was applied to determine the antifungal activity of ASS ( Schillinger & Lücke, 1989). The test microorganisms (spores for *Aspergillus niger*) with a 6–7 log load in 8 mL of soft agar (0.7%) (Malt Extract Agar) were spread on Petri dishes containing Malt Extract Agar. Then, the Petri dishes were kept at room temperature for approximately 2 h. At the end of the period, 8 mm diameter wells were opened in the Petri dishes, and plant extracts were transferred into the opened wells. Petri dishes were incubated at 30°C for 7 days and checked every day. At the end of incubation, zone diameter formation was checked for determining the presence of antifungal activity. The experiment was carried out in triplicate.

### 2.5 Physicochemical analyses

Total water‐soluble dry matter (Brix value) (*°Bx*) and pH of the samples were measured at room temperature with an Abbe refractometer (Geneq, Inc., Montreal, QC, Canada) (TSE, 1986) and potentiometric pH meter (Nel‐pH 890 model, Nel Elektronik, Türkiye) (TSE, 2001), respectively. Total acidity was determined titrimetrically and expressed as grams per one hundred grams of (g/100 g) citric acid (TSE, 2002). The total dry matter was based on a vacuum oven (Memmert V09, Memmert GmbH, Schwabach, Germany) drying method (TSE, 1996). Total sugar and invert sugar contents were determined based on the Lane–Eynon method (TSE, 2008). Total dietary fiber, protein, and fat contents were measured by a method based on the enzymatic–gravimetric principle (AOAC, 2016a), Dumas principle (Velp Dumas Nitrogen Analyzer‐NDA 701, Italy) (AOAC, 2016b), and automatic fat content analyzer **(**Velp Ser 148/6, Italy) (NMKL, 1998), respectively. Total ash content was determined by a method, which was based on removing all organic matter and water by burning the sample in a muffle furnace (NABERTHERM LV 9/11, Reagecon, Germany) at 550°C (NMKL, 2005).

A high‐performance liquid chromatography (HPLC) (Agilent 1260 Infinity, Agilent Technologies, USA) equipped with an automatic sampler and a refractive index detector (RID) was used for determining the sugar composition including fructose, sucrose, maltose, and glucose. As a mobile phase, acetonitrile:water (80:20, v/v) mixture was used. The column used was 4.6 × 250 mm in size and contained an amine‐modified filler with a particle size of 5–7 μm (Agilent Zorbax, Agilent Technologies, USA). Sugar composition (%) was calculated from the calibration curve (*R* ^2^ > .999) drawn with standard solutions containing seven different concentration levels ranging from 0.01% to 5% (AOAC, 2016c; IHC, 2009).

The amount of 5‐(hydroxymethyl) furfural (HMF) was determined by HPLC (Agilent 1260 Infinity, Agilent Technologies, USA) with a diode‐array detector (DAD) at a wavelength of 285 nm, and the results were determined as milligrams per kilogram (mg/kg). Ultrapure water:methanol (90:10, v/v) mixture was used as the mobile phase. The mobile phase flow rate was 1 mL/min. The column used was C18 and contained filler material with a size of 4.0 × 250 mm and a particle size of 5 μm (Agilent Zorbax ODS, Agilent Technologies, USA). The column oven and detector temperature was 35°C. The amount of HMF was calculated using the calibration curve (*R* ^2^ > .999) drawn with standard solutions containing different concentration levels between 0.5 and 50 mg/kg (IHC, 2009).

For elemental analysis, the samples were digested in a microwave oven (Ethos up, Milestone, Italy) with 65% nitric acid and 30% hydrogen peroxide (H_2_O_2_). Then, mineral contents were determined as milligrams per kilogram (mg/kg) by using the Inductively Coupled Plasma–Mass Spectrometer (ICP–MS) (Agilent 7500 CX, Agilent Technologies, Santa Clara, USA). Depending on the type of element investigated in the sample, three different calibration curves (*R* ^2^ > .999) at five different concentration ranges between 0.1 μg/kg and 2.5 μg/kg (for toxic elements), 0.01 mg/kg and 2.5 mg/kg (for minor elements), and 0.1 mg/kg and 25 mg/kg (for major elements) were used. If needed, an extra dilution from a sample extract was performed (NMKL, 2007).

HunterLab colorimeter (Miniscan EZ4500L, HunterLab, USA) was used to measure the color properties of the samples. In measurements; *L* ^o^ (brightness); 0 = black, 100 = white (darkness/lightness), *a* ^o^;+a red, −a green, and *b* ^o^;+b yellow, −b blue specifies color intensities. *C* ^o^ (Chroma: color saturation) ranges from 0 (dull) to 60 (vivid) and h° (Hue angle) represents tone appearance (0°, 90°, 180°, 270°, and 360°) showing red, yellow, green, blue, and red color, respectively. After these parameters were measured by a colorimeter, the following equation was used to compute the total color difference (Δ*E*), which was an indication of the changes that occurred in fortified jams during storage period compared to its traditional form:

(1) $$\Delta E=\sqrt{{\left(L^{O}-L^{1}\right)}^{2}+{\left(a^{O}-a^{1}\right)}^{2}+{\left(b^{O}-b^{1}\right)}^{2}}$$

where *L* ^o^, *a* ^o^, and *b* ^o^ represent the values of the control sample (traditional Angelica jam), and *L* ^1^, *a* ^1^, and *b* ^1^ represent the color values of the functional forms of Angelica jams (Özkan‐Karabacak et al., 2023).

### 2.6 Sensory analyses

Sensory analysis was performed during the storage period (0th days, 30th, 60th, 90th, 120th, 150th, and 180th days) with nine panelists from the academic and graduate student community of Bursa Uludağ University Food Engineering Department aged between 25 and 55 years. Jams were presented to each panelist randomly in glass cups at a recommended consumption temperature of 7°C–9°C. They were asked to score the samples, which were coded with three digits of random numbers, for appearance, consistency, odor, flavor, and general acceptability using a 9‐point hedonic scale. The proposed categories were (1) dislike extremely, (2) dislike very much, (3) dislike moderately, (4) dislike slightly, (5) neither like nor dislike, (6) like slightly, (7) like moderately, (8) like very much, and (9) like extremely (ISO, 2004).

### 2.7 Extraction of phenolics from undigested samples

For phenolic extraction, samples (2.00 ± 0.01 g) were mixed with 20 mL of aqueous methanol (75:25, v/v) acidified with formic acid (0.1%) followed by shaking at a water bath (Memmert WNB 22, Memmert, Germany) for 2 h at 20°C (Koç & Yolcı Ömeroğlu, 2019). The procedure was repeated twice. Afterward, mixtures were centrifuged (3500 rpm–10 min–4°C, Sigma 3K30) and subsequently stored at −18°C until further analysis of total antioxidant capacity (TAC), total phenolic content (TPC), and total flavonoid content (TFC).

### 2.8 In vitro digestion models

A standardized in vitro digestion model introduced by Minekus et al. (2014) was applied. This model consists of a three‐step procedure that simulates gastrointestinal (GI) digestion at the mouth, stomach, and small intestine. After each stage, 2 mL of the sample was taken. These stages included gastric digestion referred to as post‐gastric (PG), and intestinal digestion, comprising IN (indicating the material that entered the serum; the dialyzable fraction) and OUT (indicating the material that persisted in the GI tract; the nondialyzable fraction after intestinal digestion). The pH of each aliquot was adjusted to 2 and centrifuged at 23,000×*g* for 5 min at +4°C and the supernatant liquid was collected for analysis. The supernatants were stored at −20°C until analysis. Then, these samples were subjected to TAC, TPC, and TFC analyses. Analytical results of each parameter for the in vitro digested samples after intestinal digestion were compared with those obtained for the undigested ones to determine the bioaccessibility (%) of the phenolic content.

### 2.9 Total antioxidant capacity (TAC), total phenolic content (TPC), and total flavonoid content (TFC)

Total antioxidant capacity (TAC) of the samples was measured with three different assays spectrophotometrically (Shimadzu UV‐1700 spectrophotometer, Tokyo, Japan). Those assays were DPPH (1,1‐diphenyl‐2‐picrylhydrazyl) – (measured at 517 nm) (Kumaran & Karunakaran, 2006), CUPRAC (copper reducing antioxidant capacity) – (measured at 450 nm) (Apak et al., 2004), and ABTS (2,2′‐azino‐bis‐3‐ethylbenzothiazoline‐6‐sulfonic acid) – (measured at 734 nm) (Rice‐Evans et al., 1997). In all TAC assays, the results were expressed as milligrams of Trolox® equivalent (TE) per kilogram dry matter (DM) of the sample (linear range: 10–800 ppm, respectively; *R* ^2^ = .996–.999). TPC was measured using Folin–Ciocalteu reagent spectrophotometrically at 765 nm as described by Velioglu et al. (1998) and the results were expressed as milligrams of gallic acid equivalent (GAE) per kilogram dry matter (DM) of the sample (linear range: 10–400 ppm, *R* ^2^ > .999). Total flavonoid content was determined calorimetrically at 510 nm, as described by Kim et al. (2003). Results are expressed as milligrams of rutin equivalent (RE)/100 g dry matter (DM) of the sample (linear range: 10–800 ppm, *R* ^2^ > .999).

### 2.10 HPLC–DAD analysis of phenolic acids and flavonoids

Phenolic acids and major flavonoid profiles of samples were determined following the method of Capanoglu et al. (2008). Sample extracts were filtered through a 0.45‐μm membrane filter and analyzed using a Waters 2695 HPLC system with a Photodiode Array Detector (PDA) (Waters 2996) detector. A Supelcosil LC‐18 (25 cm × 4.60 mm, 5 m column, Sigma‐Aldrich, Steinheim, Germany) was used. The mobile phase consisted of solvent A, Milli‐Q water with 0.1% (v/v) trifluoroacetic acid (TFA), and solvent B, acetonitrile with 0.1% (v/v) TFA. A linear gradient was used as follows: At 0 min, 95% solvent A and 5% solvent B; at 45 min, 65% solvent A and 35% solvent B; at 47 min, 25% solvent A and 75% solvent B; and at 54 min returning to initial conditions. The flow rate was 1 mL/min. Detection was done at 280, 312, and 360 nm. Identification was based on the retention times and characteristic ultraviolet (UV) spectra. Quantification was done using external standards as well as taking the information from the literature into account. The calibration curves of polyphenol standards showed good linearity (*R* ^2^ > .99) within the established range (0.1–200 ppm (parts per million)). The limit of detection (LOD) and the limit of quantitation (LOQ) ranged from 0.1 to 0.3 mg/kg and 0.3 to 0.9 mg/kg, respectively. The results were expressed as mg/kg samples.

### 2.11 Statistical analysis

All analyses were performed in triplicate. The statistical analyses were performed using IBM SPSS Statistics 23.0 software, and the results were expressed as means ± standard deviation. The findings of the measurements were subjected to an analysis of variance (one‐way ANOVA). The presence of significant differences (*p* < .05) between means was determined using post hoc Tukey's multiple range tests.

## 3 RESULTS AND DISCUSSION

### 3.1 Characterization of the *Angelica sylvestris* L. stem

The physicochemical properties of the stem (ASS) used in Angelica jam, including total dry matter, total water‐soluble dry matter (Brix value), pH, acidity (citric acid), total protein, ash, and dietary fiber content, were obtained as 3.98 ± 0.40 g/100 g, 3.55 ± 0.25 *°Bx*, 3,97 ± 0,02, 0.32 ± 0.034 g/100 g, 1.05 ± 0.13 g/100 g, 0.40 ± 0.02 g/100 g, and 1.65 ± 0.30 g/100 g, respectively. Moreover, fat was not determined in the stem of the plant. Those results were in line with those of the previous findings of Koç and Yolcı Ömeroğlu (2019) about ASS harvested from the same location in different seasons. The *L* ^o^, *a* ^o^, *b* ^o^, Chroma, and Hue values were measured as 53.36 ± 0.07, −1.98 ± 0.02, 19.44 ± 0.09, 19.55 ± 0.08, and 95.82 ± 0.09, respectively.

The most abundant minerals were potassium (K), calcium (Ca), magnesium (Mg), and phosphorus (P) with a concentration of 845.36 ± 97.05 mg/kg, 710.58 ± 73.82, 148.04 ± 14.93 mg/kg, and 82.96 ± 9.31 mg/kg, respectively. The levels of the other element in the raw material including sodium (Na) and iron (Fe) were measured as 17.96 ± 2.06 mg/kg and 1.174 ± 0,107 mg/kg, respectively. The minor elements, including zinc (Zn), manganese (Mn), copper (Cu), chromium (Cr), cobalt (Co), bromine (Br), boron (B), and lithium (Li), were below the limit of detection (LOD) of the method specified in Table 1. Consistent with our observation, a recent study reported that ethyl alcohol extract of dried *Angelica archangelica* branch was dense with P, Mg, Ca, and K, with a range of 6422.79–130154.35 mg/kg and the level of the trace elements ranged between 0.015 and 44.99 mg/kg. Cadmium (Cd) was reported as 0.283 mg/kg, moreover the level of mercury (Hg) as a toxic element was not detected (Topal et al., 2021). Sun et al. (2020) established multielement fingerprints of mineral nutrients and toxic metals of Chinese Angelica harvested from three geographic origins in China. They found that P was the most abundant major element with a mean value of 4664.39 mg/kg, minor elements (including Zn, Mn, Cu, vanadium (V), Na, nickel (Ni), and strontium (Sr)) displayed mean values in the range of 1–50 mg/kg, and toxic elements (arsenic (As), cadmium (Cd), and lead (Pb)) ranged between 0.02 and 2.02 mg/kg.

**TABLE 1:** Physicochemical properties of the jams.

Parameter	AJ	CIAJ	CAJ
Total dry matter content, g/100 g	73.07 ± 2.75^a^	73.17 ± 2.86^a^	73.15 ± 2.87^a^
Total water‐soluble dry matter°Bx	71.80 ± 0.39^a^	72.20 ± 0.65^a^	72.70 ± 0.65^a^
pH	3.65 ± 0.09^c^	3.91 ± 0.09^a^	3.78 ± 0.09^b^
Total acidity, g/100 g (citric acid)	0.10 ± 0.00^b^	0,07 ± 0.00^b^	0.08 ± 0.00^b^
Total sugar content, %	71.00 ± 3.00^a^	71.30 ± 3.10^a^	71.90 ± 3.10^a^
Invert sugar content, %	39.3 ± 1.40^b^	49.20 ± 1.70^a^	49.60 ± 1.70^a^
Sugar composition %			
Glucose	20.50 ± 1.10^b^	24.90 ± 1.30^a^	25,10 ± 1,20^a^
Fructose	18.20 ± 0.90^b^	23.80 ± 1.10^a^	24,00 ± 1,20^a^
Sucrose	32.40 ± 2.40^a^	22.50 ± 1.70^b^	22.00 ± 1.70^b^
Dietary fiber content, g/100 g	0.70 ± 0.08^b^	0.80 ± 0.03^b^	0.90 ± 0.05^b^
HMF, mg/kg	91.00 ± 5.20^b^	101.80 ± 5.20^a^	105.50 ± 5.40^a^
P	7.57 ± 0.85^c,d^	18.96 ± 2.13^b,c^	28.06 ± 3.15^b^
Na	36.05 ± 4.14^a^	9,45 ± 1,08^c^	12.18 ± 1.39^b,c^
Mg	24.00 ± 2.41^b,c^	30.76 ± 3.09^b,c^	43.28 ± 4.35^b^
K	80.49 ± 9.24^d^	213.068 ± 24.46^c^	388.67 ± 44.62^b^
Ca	120.07 ± 12.48^b^	111.10 ± 11.54^b^	135.00 ± 14.03^b^
Fe	0.52 ± 0.05^c^	1.14 ± 0.10	0.81 ± 0.07^b^
Zn, Mn, Cu, Cr, Co, Br, B, Li^a^	<LOD	<LOD	<LOD

Abbreviations: AJ, Anjelica jam; CIAJ, Angelica jam fortified with cinnamon extract; CAJ, Angelica jam fortified with carob extract.

^a^ LOD of those elements are 0.221 mg/kg, 0.554 mg/kg, 0.209 mg/kg, 0.220 mg/kg, 0.210 mg/kg, 0.188 mg/kg, 0.496 mg/kg, and 0.554 mg/kg, respectively. Different lower case letters indicate significant differences in the same row (*p* < .05).

Total antioxidant capacity of ASS ranged between 140.66 ± 12.9‐ and 351.25 ± 25.50 mg TE/100 g DM, measured with spectrophotometric assays with an order of ABTS > CUPRAC > DPPH. Total flavonoid and phenolic contents were 1.94 ± 0.05 mg RE/100 g DM and 3.55 ± 0.29 mg GAE/100 g DM, respectively. Karaçelik et al., 2022 reported the phenolic content of the methanolic extract prepared from AS stem harvested from Giresun province of Turkey as 543.91 ± 6.33 GAE μg/mL. Topal et al. (2021) revealed that ethanolic extract of *Angelica archangelica* branch and leaf contained total phenolic compound with a range of 53.50 and 55.50 μg/mg GAE. The difference in the total antioxidant capacity and amount of total phenolic compound can be attributed to the geographic origin, in addition to efficiency of extraction solvent and the part of the plant extracted.

According to the antifungal activity results, clear inhibition zones were not observed on the wells against *Saccharomyces cerevisiae, Schizosaccharomyces pombe*, and *A. niger*. Even though ASS did not show any fungicidal effect in the current study, the other studies reported in the literature revealed the antibacterial effects of AS, including *Enterococcus faecium*, *Listeria monocytogenes*, *Bacillus subtilis*, *Staphylococcus epidermidis*, *Staphylococcus aureus*, and *Pseudomonas aeruginosa* (Canli et al., 2016; Han & Guo, 2012; Oral et al., 2008). Canli et al. (2016) reported that ethanol extract of AS showed stronger antimicrobial activity against *E. faecium*, *L. monocytogenes*, *B. subtilis*, *S. epidermidis*, and *S. aureus* and more than the other 13 strains including *Candida albicans* as a fungus. This explains the fact that antimicrobial effects of AS against bacterial strains are stronger compared to its effect against fungus. Moreover, *A. nige*r has been known as the most stable microorganism and has resisted too many antimicrobial compounds (Korukluoğlu et al., 2006; Quiroga et al., 2001). Those facts can be attributed to our observations related to the non‐fungicidal effects of AS ethanol extracts.

### 3.2 Physicochemical properties of the jams

The common physicochemical properties of the jams are shown in Table 1. Total water‐soluble dry matter (Brix value) is one of the quality criteria in jam and similar products, and determines the amount of sugar, acid, and pectin to be added to the recipe. In the scope of this study, Brix value ranged in between 71.80 and 72.70 *°Bx*, which is in line with the range stated in the literature as 65–79 *°Bx* (Tamer, 2012; Kamiloglu et al., 2015; Tomas et al., 2017) and meets the requirement of traditional jam categories (Anonymous, 2006). The recent study reported by Koç and Yolcı Ömeroğlu (2019) demonstrated that Angelica jam produced traditionally in Bursa satisfies the requirements of the “extra traditional jam” category with its 45% fruit content and 72 *°Bx* value.

Since the total dry matter of a jam varies according to the amount of fruits and sugar included in the recipe, it was observed that there was no significant difference (*p* > .05) between the total dry matter content of the Angelica jams (AJ, CIAJ, and CAJ).

pH values changed between 3.65 and 3.91 (Table 1). This range was consistent with the pH value reported by Koç and Yolcı Ömeroğlu (2020) for Angelica jam produced from the raw material harvested at different seasons. On the other hand, the range is slightly higher than the maximum value stated in the legislation as 3.5 (Anonymous, 2006). It is very obvious that addition of the extract for increasing the functional properties of the Angelica jam increased the pH values of the final product slightly. Kim et al. (2014) observed that addition of cinnamon powder to cake increased the pH of the final product due to the cinnamic acid in cinnamon. Proper gel formation is provided by keeping the pH value at the optimum level. Even though pH range of a jam depends on the varieties of the fruit (Tamer, 2012), it can be made possible to decrease pH value by adding proper amount of organic acid (Cemeroğlu et al., 2003).

The sugar content of the Angelica jams (AJ, CIAJ, CAJ) ranged between 71.00% and 71.90%. There was no significant difference statistically between Angelica jams (*p* > .05) in terms of total sugar content. Addition of sugar during jam production provides increased shelf life to the fruit by decreasing water activity in addition to increasing of the consistency of the final product, which is an important point for consumer perception. Depending on the type of fruit and vegetables, the range for sugar content of jams changed between 65.20% and 73.50% in the studies reported in the literature (Tamer, 2012). Total sugar content of cinnamon and carob was reported as 2.17% and 32%–67% (Pazir & Alper, 2018; Yalım‐Kaya & Özdemir, 2015), respectively. The amount of extract added into Angelica jam was limited to 1% based on the sensory acceptance level and the ASS did not contain sugar; therefore, the sugar content of the jams s originated from the sugar added at the processing step. Therefore, sugar substitutes and thickening agents should be used for further studies to develop low‐calorie Angelica jam.

Invert sugar content and sugar composition of functional formulations of Angelica jams (CIAJ, CAJ) were significantly different than those of Angelica jam (AJ) (*p* < .05). The difference can be attributed to the extra heating process in the production of functional formulations after addition of the cinnamon and carob extracts. The sugar composition of AJ is similar to findings reported in the literature (Tamer, 2012). During the production and storage of jams, sucrose undergoes inversion depending on temperature, time, and pH, and then invert sugar is formed.

Since jams do not contain protein‐based raw materials, the protein content of Angelica jams (AJ, CIAJ, CAJ) remained below the method of detection limit (<0.05% N). Moreover, fat content was not determined in the jams. Based on the previous studies, fat content of jams is very low due to the fact that their raw materials did not contain high fat content (Gupta et al., 2016). It was determined that total dietary fiber content of Angelica jams ranged from 0.70 to 0.90 g/100 g. Dietary fiber is defined as “carbohydrate polymers with three or more monomeric units that are not digested or absorbed in the human small intestine” (EU, 2011). Cinnamon and carob are a good source of dietary fiber with a range of 11–53.10 g/100 g and 11–40 g/100 g, respectively. Due to the fortification of Angelica jam with their extracts, functional formulations may not contribute more to the daily dietary fiber intake of a consumer (Gupta et al., 2016; Pazir & Alper, 2018; Yalım‐Kaya & Özdemir, 2015). Besides, our results are in line with the findings reported in the literature as 0.30–1.41 g/100 g (Gupta et al., 2016).

The HMF content of functional formulations of Angelica jams (CIAJ, CAJ) was slightly higher than that of AJ due to the extra heating process (Table 1). Our findings are in line with those of the previous studies in the literature with a HMF value range of 69.22–1094.10 mg/kg (Şengül et al., 2018). The high values were attributed to high temperature and prolonged cooking period. During the production and storage of foods, Maillard reactions can take place between sugars and amino acids with the effect of heat. HMF, which is an important intermediate product of Maillard reactions, causes a decrease in nutritional values of foods, undesirable taste and color changes, and deterioration of quality. Moreover, HMF content is limited in many products due to its carcinogenic effect (Choudhary et al., 2021). Therefore, it is recommended that, production and storage should be carried out under controlled temperature conditions, including cooking under vacuum to reduce the HMF content of jams.

Change of mineral content of Angelica jams by adding different types of extract is shown in Table 1. K, Ca, and Mg are well‐known macro elements and have an important role in human health, including contraction of muscle, regulation of blood pressure, development of skeletal system, the metabolism of carbohydrates and fats, protecting the bone structure, etc. (Topal et al., 2021). Likewise, as regards the elemental composition in raw material, K and Ca are the most abundant minerals in the jams. In addition, Mg, P, Na, and Fe, and K were observed in the jams in descending order. Processing of ASS into jam decreased the elemental composition of the final product. On the other hand, addition of cinnamon and carob extracts to Angelica jam increased mineral content in the final product. This can be attributed to the rich elemental content of the cinnamon and carob reported in the literature (Al‐Numair et al., 2007; Sontakke et al., 2018).

### 3.3 Changes of color and sensory properties of the jams through the shelf life period

The change of the color parameters of the Angelica jams throughout the 6‐month storage period is presented in Table 2. Based on the color parameters, it was concluded that AJ and ASS were yellow in color. This is in line with our previous findings (Koç & Yolcı Ömeroğlu, 2019). Moreover, it was observed that the *L* ^o^ value decreased by 41.99% in jam processing, indicating that the darkness increased. It was observed that while the *a* ^o^ value increased, *b* ^o^, *C* ^o^, and *h* ^o^ values decreased throughout the jam processing. Color, aroma, and texture are important quality criteria that ensure the acceptability of foods. The color characteristics of foods indicate quality changes during processing and storage. Studies have shown that the *L* ^o^ value is a measure of caramelization, and this darkening was explained by the denaturation of color pigments at high temperatures (Tamer, 2012). The *L* ^o^, *a* ^o^, *b* ^o^, *C* ^o^, and *h* ^o^ values were measured as 0.11 ± 0,02, 0.09 ± 0.01, −0.07 ± 0.01, 0.12 ± 0.15, and 320.75 ± 0.65, respectively, for cinnamon extract. The corresponding values obtained for carob extract were measured as 0.02 ± 0.02, 0.16 ± 0.01, −0.12 ± 0.01, 0.21 ± 0.02, and 323.39 ± 0.54, respectively. Therefore, color parameters of the Angelica jam and its functional formulations were significantly different from each other (*p* < .05). *L* ^o^ values of Angelica jams fortified with extracts of cinnamon (CIAJ) and carob (CAJ) were found to be 11.04 and 5.26, respectively. Although the amount of cinnamon and carob extracts added to angelica jam for functionalization purposes was limited, it increased the darkness of the jams. Kim et al. (2014) revealed that an increase in cinnamon powder ratio in rice cake resulted in a decrease in *L* ^o^, and an increase in *a* ^o^ values, and *b* ^o^ values increased with the addition of 1% cinnamon powder and decreased in rice cakes containing more than 1% cinnamon powder. In line with our findings, Baykal et al. (2018) reported that the *L* ^o^ value of goat milk powders enriched with cinnamon was lower than that of plain goat milk powder, and the *a* ^o^ and *b* ^o^ values of goat milk powders enriched with cinnamon and carob were generally higher compared to the *a* ^o^ and *b* ^o^ values of plain milk powder.

**TABLE 2:** Color properties of the raw materials and jams.

Color parameter	*L* ^o^	*a* ^o^	*b* ^o^	Chroma (*C* ^o^)	Hue (*h* ^o^)
ASS	53.36 ± 0.07^a^	−1.98 ± 0.02^e^	19.44 ± 0.09^a^	19.55 ± 0.08^a^	95.82 ± 0.09^b^
AJ	23.32 ± 0.43^b^	3.46 ± 0.08^b^	9.94 ± 0.04^b^	10.53 ± 0.15^b^	89.43 ± 0.85^c^
CIAJ	11.04 ± 0.56^c^	10.46 ± 0.40^a^	9.68 ± 0.70^b^	12.93 ± 2.43^b^	42.73 ± 1.28^d^
CAJ	5.26 ± 0.22^d^	−0.45 ± 0.09^d^	5.77 ± 0.41^c^	5.80 ± 0.41^c^	94.52 ± 1.09^b^
CIE	0.11 ± 0.02^e^	0.09 ± 0.01^c^	−0.07 ± 0.01^d^	0.12 ± 0.015^d^	320.75 ± 0.65^a^
CAE	0.02 ± 0.02^e^	0.16 ± 0.01^c^	−0.12 ± 0.01^d^	0.21 ± 0.02^d^	323.39 ± 0.54^a^

*Note*: Different lowercase letters indicate significant differences in the same column (*p* < .05).

Abbreviations: AJ, Anjelica jam; ASS, Angelica sylvestris stem; CAE, carob extract; CAJ, Angelica jam fortified with carob extract; CIAJ, Angelica jam fortified with cinnamon extract; CIE, cinnamon extract.

Regarding the effect of storage period on the color parameters of the jams, it was observed that no statistical difference was found in the color values of the control samples (AJ) during the 6‐month shelf life (*p* > .05) (Table 3). Although there was a statistically significant (*p* < .05) increase in the *L* ^o^ value of CAJ, no significant (*p* > .05) increase was observed for the other values. In the last month of the storage period, a statistical difference was observed between the *L* ^o^ values of CIAJ compared to those obtained in the other periods (*p* < .05). However, there was no statistically significant change in the *h* ^o^ value, which indicates the color tone. In line with our findings, previous studies have revealed that chemical modifications of phenolic compounds during the storage period caused changes in color values (Abuduaibifu & Tamer, 2019). Aslanova et al. (2010) stated that color changes during shelf life depend on more than one reaction, and that non‐enzymatic darkening reactions and changes in pigment structure are also effective. In another study, a decrease in *L* ^o^ and *b* ^o^ values throughout the storage was attributed to both the formation of HMF through the Maillard reaction and oxidation of vitamin C (Damiani et al., 2017). On the other hand, Holzwarth et al. (2013) recommended the cold storage of jams at 4°C to preserve the color characteristics of the jams.

**TABLE 3:** Changes of color parameters of Angelica jams throughout the storage period.

	1. Month	2. Month	3. Month	4. Month	5. Month	6. Month
L*						
AJ	22.53 ± 0.43 ^a^	22.49 ± 0.39^a^	22.61 ± 0.46^a^	22.55 ± 0.21^a^	22.18 ± 0.20^a^	23.32 ± 0.64^a^
CIAJ	11.04 ± 0.56^b,c^	10.67 ± 0.11^c^	11.46 ± 0.10^b^	11.01 ± 0.24^b,c^	10.91 ± 0.02^b,c^	12.68 ± 0.08^a^
CAJ	5.26 ± 0.22^c^	5.22 ± 0.053^c^	6.36 ± 0.11^b^	5.82 ± 0.05^b,c^	5.81 ± 0.17^b,c^	8.85 ± 0.86^a^
a*						
AJ	3.36 ± 0.09^a^	3.37 ± 0.11^a^	3.38 ± 0.06^a^	3.33 ± 0.10^a^	3.37 ± 0.10^a^	3.47 ± 0.09^a^
CIAJ	10.46 ± 0.40^b^	10.09 ± 0.11^b^	11.91 ± 0.21^a^	10.21 ± 0.16^b^	9.16 ± 0.03^c^	9.45 ± 0.13^c^
CAJ	−0.45 ± 0.09^a^	−0.56 ± 0.09^a^	−0.55 ± 0.09^a^	−0.52 ± 0.02^a^	−0.68 ± 0.16^a^	−0.47 ± 0.29^a^
*b*						
AJ	9.94 ± 0.06^a^	9.90 ± 0.09^a^	9.89 ± 0.06^a^	9.89 ± 0.03^a^	9.89 ± 0.03^a^	9.91 ± 0.04^a^
CIAJ	9.68 ± 0.7^a.b^	9.49 ± 0.19^b^	10.55 ± 0.22^a^	8.01 ± 0.13^c^	7.91 ± 0.21^c^	8.55 ± 0.13^c^
CAJ	5.72 ± 0.41^a^	5.83 ± 0.17^a^	6.42 ± 0.29^a^	5.82 ± 0.13^a^	5.89 ± 0.06^a^	6.68 ± 1.12^a^
C*						
AJ	10.59 ± 0.20^a^	10.69 ± 0.12^a^	10.62 ± 0.21^a^	10.64 ± 0.09^a^	10.60 ± 0.20^a^	10.53 ± 0.15^a^
CIAJ	12.93 ± 2.43^b^	13.85 ± 0.18^a,b^	15.91 ± 0.31^a^	12.97 ± 0.21^b^	12.11 ± 0.14^b^	12.77 ± 0.18^b^
CAJ	5.80 ± 0.41^a^	5.95 ± 0.42^a^	6.41 ± 0.29^a^	5.84 ± 0.12^a^	6.16 ± 0.08^a^	6.71 ± 1.09^a^
*H* ^0^						
AJ	89.43 ± 1.66^a^	89.38 ± 1.73^a^	89.59 ± 1.29^a^	89.37 ± 2.29^a^	89.61 ± 1.59^a^	89.43 ± 0.84^a^
CIAJ	42.73 ± 1.28^a^	43.25 ± 0.49^a^	41.52 ± 0.09^a,b^	38.13 ± 0.22^c^	40.77 ± 0.75^b^	42.01 ± 0.20^a,b^
CAJ	94.52 ± 1.09^a^	94.86 ± 3.92^a^	94.35 ± 1.96^a^	95.09 ± 0.32^a^	95.76 ± 03.42^a^	94.40 ± 3.49^a^
Δ*E*						
AJ	‐	‐	‐	‐	‐	‐
CIAJ	13.53 ± 0.29^a,b^	13.61 ± 0.32^a,b^	14.05 ± 0.34^a^	13.56 ± 0.31^a,b^	13.15 ± 0.12^b,c^	12.31 ± 0.44^c^
CAJ	18.17 ± 0.59^a^	18.17 ± 0.38^a^	17.07 ± 0.52^a^	17.64 ± 0.25^a^	17.33 ± 0.24^a^	15.36 ± 0.83^b^

*Note*: Different lowercase letters indicate significant differences in the same row (*p* < .05).

Abbreviations: AJ, traditional Anjelica jam; CAJ, jam fortified with carob extract; CIAJ, jam fortified with cinnamon extract.

Additionally, it was observed that the Δ*E* values, which express the difference of functional formulations of Angelica jams (CIAJ, CAJ) from the control (AJ), decreased significantly (*p* < .05) throughout the storage period at 4°C. Even though reactions that caused color change increased at the 6th month of the period with a rate of 1.22% in CIAJ and 2.81% in CAJ, the rate of change was obviously low. This can be attributed to the cold storage of jams, as indicated by Kamiloglu et al. (2015).

**FIGURE 2:**
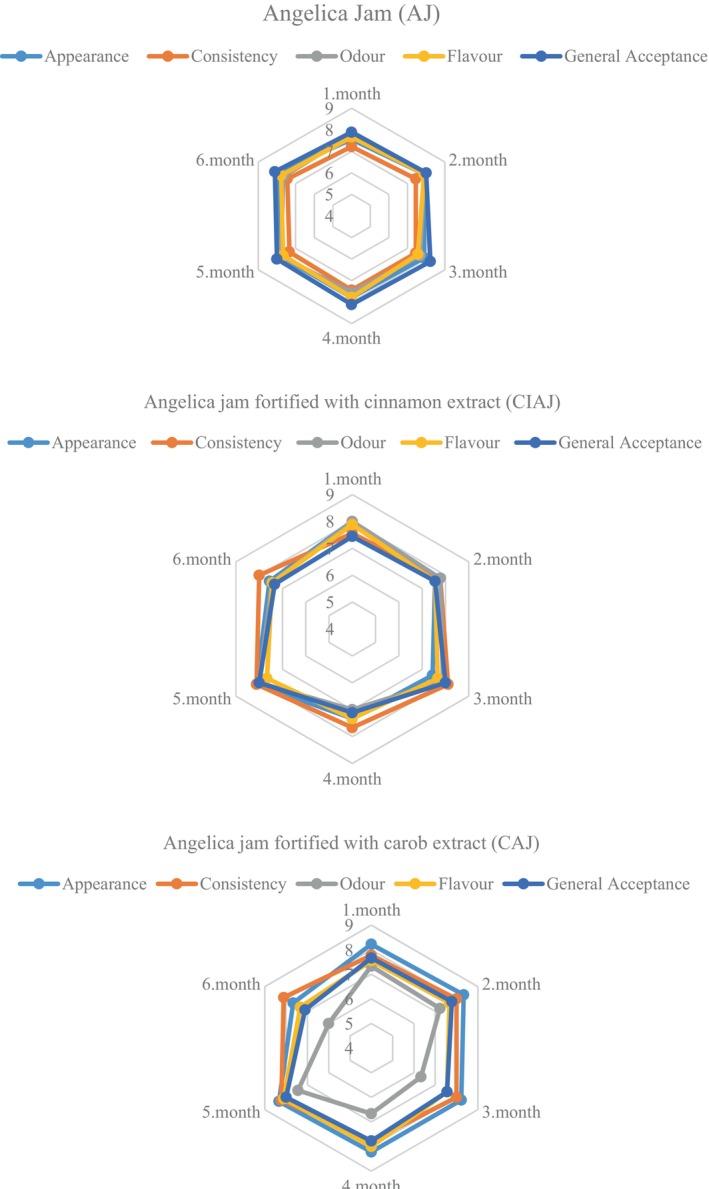
Changes of sensorial quality of Angelica jams throughout the storage period.

Regarding change of sensory properties during the cold storage period, it was observed that all parameters, except the odor parameter of CAJ, ranged between 7.11 and 8.33 without any significant difference throughout the storage (*p* < .05) (Figure 2). On the other hand, it was stated that after the 3rd month of the shelf life, the odor of carob became increasingly dominant and therefore a decrease was observed in the scoring compared to the previous months. Likewise, Jain et al. (2011) stated that the decrease in the general acceptability of the jam of guava and papaya with addition of gooseberries could be related to the increase in storage time. When the general acceptability of all jams was examined, it was observed that AJ, CIAJ, and CAJ obtained a score range of 7.89–8.22, 7.33–7.89, and 7.11–8.00, respectively. Future studies should be concentrated on the detailed physicochemical and microbiological analysis during shelf life.

### 3.4 Total antioxidant capacity, total phenolic content, and flavonoid content and their bioaccessiblity

The changes in the total antioxidant capacity of Angelica jams and raw materials (*A. sylvestris* stem, carob extract, and cinnamon extract) were determined using three different assays: ABTS, CUPRAC, and DPPH (Table 4 and Table 5).

**TABLE 4:** Changes in the total antioxidant capacity (ABTS, CUPRAC, and DPHH assays), total phenolic and flavonoid content of Angelica jams during in vitro gastrointestinal digestion.

	Undigested	PG	IN	OUT	Bioaccessibility %
Total antioxidant capacity‐ ABTS (mg TE/100 g DM)					
ASS	351.25 ± 25.50^d,A^	942.24 ± 39.37^c,A^	2967.55 ± 328.70^b,A^	5842.35 ± 252.08^a,A^	844
AJ	96.68 ± 11.30^c,D^	43.49 ± 2.94^d,C^	127.89 ± 8.51^b,C^	258.21 ± 8.10^a,C^	132
CIAJ	193.11 ± 16.06^b,C^	50.09 ± 2.23^d,C^	125.37 ± 11.70^c,C^	227.08 ± 20.37^a,D^	64
CAJ	248.49 ± 17.78^b,B^	84.54 ± 5.36^d,B^	162.97 ± 21.48^c,B^	285.34 ± 17.16^a,B^	65
Total antioxidant capacity‐CUPRAC (mg TE/100 g DM)					
ASS	166.06 ± 24.31^a,B^	42.75 ± 4.46^c,B^	59.68 ± 12.39^c,A^	108.76 ± 10.49^b,A^	36
AJ	41.78 ± 4.13^a,D^	10.028 ± 0.35^b,C^	3.63 ± 0.56^c,C^	4.81 ± 0.55^b,c,D^	9
CIAJ	106.08 ± 16.16^a,C^	7.69 ± 0.48^b,C^	3.14 ± 0.48^b,C^	6.28 ± 0.62^b,D^	3
CAJ	273.11 ± 29.63^a,A^	55.75 ± 1.98^b,A^	3.00 ± 0.32^c,C^	9.40 ± 0.67^c,C^	1
Total antioxidant capacity‐DPPH (mg TE/100 g DM)					
ASS	140.66 ± 12.94^d,A^	7325.48 ± 71.38^a,A^	2449.20 ± 74.69^b,A^	2227.95 ± 224.870^c,A^	1741
AJ	22.44 ± 0.58^c,B^	348.48 ± 22.07^a,B^	109.91 ± 4.50^b,C^	118.72 ± 9.56^b,C^	489
CIAJ	35.33 ± 1,41^c,B^	336.88 ± 2.74^a,B^	113.06 ± 7.39^b,C^	117.64 ± 7.21^b,C^	319
CAJ	131.19 ± 1.65^c,A^	322.38 ± 8.82^a,B^	119.21 ± 7.74^d,C^	143.59 ± 4.96^b,B^	90
Total phenolic content (mg GAE/100 g DM)					
ASS	3.55 ± 0.29^d,D^	197.11 ± 18.37^c,A^	328.59 ± 16.23^b,A^	435.52 ± 33.02^a,A^	925
AJ	6.13 ± 0.66^c,C^	10.31 ± 1.22^b,C^	13.11 ± 1.18^b,B^	22.03 ± 3.03^a,B^	213
CIAJ	11.64 ± 0.80^b,B^	11.35 ± 0.84^b,C^	12.44 ± 0.61^b,B^	22.87 ± 1.65^a,B^	106
CAJ	26.33 ± 1.44^a,A^	17.85 ± 2.56^c,B^	13.45 ± 0.89^d,B^	21.46 ± 1.61^b,B^	51
Total flavonoid content (mg TE/100 g DM)					
ASS	1.94 ± 0.05^c,C^	205.82 ± 2.60^a,A^	5.89 ± 1.74^b,c,A^	9.47 ± 1.82^b,A^	302
AJ	1.71 ± 0.01^b,C^	13.51 ± 2.59^a,B^	0.19 ± 0.04^b,B^	0.60 ± 0.06^b,B^	10
CIAJ	7.17 ± 0.86^a,A^	4.33 ± 0.79^b,C^	0.37 ± 0.08^c,B^	0.21 ± 0.01^c,B^	5
CAJ	6.47 ± 0.56^a,A^	1.71 ± 0.14^b,C^	0.20 ± 0.11^c;B^	0.36 ± 0.27^c,B^	3

*Note*: Data represent average values ± standard deviation of three independent samples. All contents are expressed as milligrams (mg) per 100 g dry weight (dw). Different capital letters in the rows represent statistically significant differences throughout digestion (*p* < .05). Different lowercase letters in the coloumn represent statistically significant differences between jams (*p* < .05).

Abbreviations: AJ, traditional Anjelica jam; ASS, *Angelica sylvestris* stem; CAJ, jam fortified with carob extract; CIAJ, jam fortified with cinnamon extract; IN, dialyzed fraction after intestinal digestion; OUT, non‐dialyzed fraction after intestinal digestion; PG, antioxidants and phenolics remaining after gastric digestion.

**TABLE 5:** Changes in the total antioxidant capacity (ABTS, CUPRAC, and DPHH assays), total phenolic and flavonoid content of extracts during in vitro gastrointestinal digestion.

	Undigested	PG	IN	OUT	Bioaccessibility %
Total antioxidant capacity‐ ABTS (mg TE/100 g DM)					
CIE	37493.47 ± 932.72^a,A^	13881.40 ± 139.22^a,D^	20625.10 ± 939.15^b,C^	31872.50 ± 105.77^a,B^	55
CAE	38457.97 ± 114.97^a,A^	12144.58 ± 38.21^a,D^	22153.21 ± 170.34^a,C^	27655.21 ± 87.70^b,B^	58
Total antioxidant capacity‐CUPRAC (mg TE/100 g DM)					
CIE	28646.49 ± 4366.36^a,A^	1695.55 ± 33.74^a,B^	667.75 ± 60.19^b,B,C^	2238.10 ± 170.95^b,B^	2
CAE	22447.35 ± 1718.85^b,A^	1153.65 ± 13.16^b,B,C^	1079.33 ± 149.87^a,B^	2427.22 ± 222.74^a,B^	5
Total antioxidant capacity‐DPPH (mg TE/100 g DM)					
CIE	8760.23 ± 305.57^a,C^	19093.84 ± 151.46^a,A^	11463.74 ± 501.83^b,AB^	19510.42 ± 1305.06^b,A^	131
CAE	8281.18 ± 486.37^b,B^	16681.93 ± 144.52^a,B^	32735.71 ± 817.77^a,A^	37628.75 ± 1341.50^a,A^	395
Total phenolic content (mg GAE/100 g DM)					
CIE	1400.97 ± 48.65^a,C^	3582.20 ± 653.57^b,B^	1993.11 ± 178.29^b,C^	4650.02 ± 323.63^a,A^	142
CAE	1436.73 ± 86.34^a,C^	3799.22 ± 164.48^a,A,B^	2730.34 ± 84.57^a,B^	4273.52 ± 405.93^a,A^	190
Total flavonoid content (mg TE/100 g DM)					
CIE	1881.99 ± 111.42^a,A^	387.00 ± 16.16^b,B^	325.00 ± 20.46^a,B^	451.08 ± 41.33^b,B^	13
CAE	424.17 ± 79.43^b,B^	987.16 ± 139.26^a,A^	351.68 ± 48.39^a,B^	496.01 ± 12.38^a,B^	83

*Note*: Data represent average values ± standard deviation of three independent samples. All contents are expressed as mg per 100 g dry weight (dw). Different capital letters in the rows represent statistically significant differences throughout digestion (*p* < .05). Different lowercase letters in the coloumn represent statistically significant differences between extract (*p* < .05).

Abbreviations: CAE, carob extract; CIE, cinnamon extract; IN, dialyzed fraction after intestinal digestion; OUT, non‐dialyzed fraction after intestinal digestion; PG, post gastric, i.e., antioxidants and phenolics remaining after gastric digestion.

The highest antioxidant capacity value of Angelica jam and its functional formulations with addition of carob and cinnamon extracts was obtained as 96.68 ± 11.30 mg TE/100 g DM, 248.49 ± 17.78 mg TE/100 g DM, and 193.11 ± 16.06 mg TE/100 g DM, respectively, with the ABTS method. Among different assays, the highest antioxidant capacity values were obtained with the ABTS method. This can be explained by the measurement capacity of ABTS method both for aqueous radicals and lipid peroxyl radicals (Koç & Yolcı Ömeroğlu, 2020). Moreover, ABTS radical is stable in a wide pH range. The DPPH radical is an important limitation in the analysis of hydrophilic antioxidants because it can only dissolve in organic media (especially in alcohol media) but not in aqueous media. Since small molecules reach radicals more easily, they have higher antioxidant capacity values. The CUPRAC reagent, which is a fast method to oxidize thiol‐type antioxidants, has a lower electrode potential, therefore simple sugars and citric acid that will cause interference are not oxidized with this reagent (Koç & Yolcı Ömeroğlu, 2020). This ensures reliable results for sugary matrices such as jam. Therefore, it is convenient to use more than one assay for measuring antioxidant capacity and moreover chromatographic methods should be applied to determine phenolic compounds (Durmus et al., 2020; Kamiloglu et al., 2015).

It was observed that the total antioxidant capacity of AAS decreased by 72%–85% with the heat treatment applied during jam processing. The same tendency was observed with all three antioxidant assay methods. Our results were in consistent with the findings reported by previous studies (Kamiloglu et al., 2015; Suna et al., 2023). Kamiloglu et al. (2015) stated that the decrease in the total antioxidant capacity is due to the destruction of those organic compounds and formation of non‐antioxidant forms during jam processing. The highest antioxidant capacity values of carob and cinnamon extracts were obtained by the ABTS assay as 37493.47 ± 932.72 mg TE/100 g DM and 38457.97 ± 114.97 mg TE/100 g DM, respectively. While the antioxidant capacity values of the Angelica jams were statistically different from each other (*p* < .05), CAJ had the highest antioxidant capacity value, followed by CIAJ. These results can be associated with the high antioxidant capacities of carob and cinnamon extracts, which were also reported in previous studies in the literature (Stavrou et al., 2018). Therefore, fortification of traditional Angelica jam with cinnamon and carob extracts increased their antioxidant capacities with a ratio changing between 57%–99% and 157%–553%, respectively. In the same manner, Rababah et al. (2011) reported that fortification of fruit juice with carob powder increased the total phenolic content and total antioxidant capacity of the product significantly. Moreover, the other studies reported in the literature revealed the same findings for functional fermented drinks and cakes fortified with cinnamon (Kim et al., 2014; Setiyoningrum et al., 2019).

In line with the findings on total antioxidant capacity of the Angelica jams, jam processing decreased the total flavonoid content of AJ. There are also other studies reported in the literature which revealed that during the processing of fruits into jam and marmalade, deterioration in the cell structure and the sensitivity of the raw material to non‐enzymatic oxidation cause a decrease in phenolic components (Tomas et al., 2017). On the other hand, total phenolic content of the Angelica jams increased significantly after jam processing. This can be attributed to the limited selectivity of the analytical method. Folin–Ciocalteu method can measure ascorbic acid, citric acid, simple sugars, and some amino acids, in addition to phenolic compounds in the sample (Koç & Yolcı Ömeroğlu, 2020). Fortification of AJ with cinnamon and carob extracts enhanced significantly the total phenolic and flavonoid content of the final product as expected based on the experimental results shown in Table 4. This is due to the higher TPC and TFC content of the extracts, as shown in Table 5.

Correlation coefficients (*R* ^2^) between spectrophotometric analyses are given in Table 6. A moderate–good linear relationship was observed between total phenolic substance and total flavonoid substance (*R* ^2^ = .625). Among the three different antioxidant capacity measurement assays used, the highest correlation was found between ABTS and DPPH methods (*R* ^2^ = .9999). Except for the flavonoid method, a good correlation was also detected between total antioxidant capacity and total phenolic substance (*R* ^2^ = .9751–.999). These results show that phenolics contribute significantly to the antioxidant capacities of the raw materials, extracts, and jam samples investigated. Another study in the literature found a positive correlation between total phenolic substances and total antioxidant capacity (Suna et al., 2023).

**TABLE 6:** Pearson's correlation coefficients between TPC, TFC, and TAC.

Analyses	ABTS	CUPRAC	DPPH	Phenol	Flavonoid
TAC–ABTS	1.000				
TAC–CUPRAC	0.9751	1.000			
TAC–DPPH	0.9976	0.9876	1.000		
TPC	0.9999	0.9751	0.9975	1.000	
TFC	0.625	0.7696	0.6713	0.625	1.000

Bioaccessibility refers to the proportion of a biocompound consumed in the diet that becomes available for absorption through the epithelial layer of the gastrointestinal tract. For successful absorption, these biocompounds need to be liberated from the food matrix or nanotransporters and subsequently solubilized within association micelles. The experimental assessment of bioaccessibility has proven effective through the utilization of the in vitro method (Dima et al., 2020). In vitro bioaccessibility studies serve as a preliminary step before in vivo studies, providing a cost‐effective and ethical means of assessing the potential health benefits of dietary components. While in vitro methods have their limitations, such as the simplified representation of complex physiological processes, they remain a valuable tool in the early stages of evaluating the fate of biocomponents during digestion (Minekus et al., 2014). The TPC of Angelica jams (AJ, CAJ, CIAJ) and the raw materials including ASS, CAE, and CIE increased by more than 100% during the gastric digestion due to the interaction of bioactive compounds with enzymes under the pH conditions of the system for an incubation period of 2 h. In line with our findings, Suna et al. (2023) reported an increase in TPC of marmalades during gastric (381.88%–509.32%) and intestinal (425.46%–642.77%) digestion compared to the undigested extracts. Kamiloglu (2019) attributes this to the fact that phenolic components are effectively released from foods during gastric digestion, while maintaining their stability due to environmental conditions. Moreover, it was related to the false positive interactions of Folin–Ciocalteu reagent with other analytes including sugars and citric acids.

Additionally, the amounts of TFC and TAC measured by the DPPH assay increased significantly after post‐intestinal digestion compared to those f obtained from the undigested sample. Therefore, the recovery of total bioaccessible phenols was much higher than 100%. That finding was attributed to the liquids and enzymes in the digestion conditions in intestine, the contact time of foods with it, the release of bioactive components from foods throughout the intestinal digestion followed by their rapid absorption by intestinal micelle (Tomas et al., 2017). Moreover, Ferri et al. (2013) stated that the DPPH method gave false positive results in acidic environments, and that the most appropriate pH range was pH 4–8.

While the bioaccessibility of antioxidant substances in Angelica jams varied between 64% and 132% when measured by the ABTS, it ranged between 1.09% and 18.89% when measured by the CUPRAC. A similar trend was observed for both cinnamon (CIE) and carob extracts (CAE). Tomas et al. (2017) obtained similar results in black mulberry marmalade. Kamiloglu (2019) indicated that the wide range of bioaccessibility values (%) was due to the difference in release mechanism of bioactive components from the matrix during digestion. The total amount of flavonoids was found to be negligible in post‐intestinal digestion.

### 3.5 Phenolic composition

The major phenolic compounds of initial, post‐gastric, intestinal IN and OUT phases of ASS, cinnamon extract (CIE), carob extract (CE), traditional angelica jam (AJ), angelica jam fortified with cinnamon extract (CIAJ), and with carob extract (CAJ) were detected by HPLC–DAD analysis. A comparison of the phenolic profiles of samples is shown in Table 7.

**TABLE 7:** Phenolic profiles of initial gastric and intestinal phases of samples after in vitro GI digestion.

	Undigested	PG	IN	OUT
Caffeic acid (mg/kg DM)				
ASS	295.1 ± 64.0	173.2 ± 23.5	122.1 ± 49.4	122.0 ± 19.8
CIE, CAE	<LOD	<LOD	<LOD	<LOD
AJ	4.2 ± 1.5	0.9 ± 0.7	<LOD	4.7 ± 0.1
CIAJ	4.7 ± 1.6	1.4 ± 0.1	<LOD	<LOD
CAJ	7.0 ± 0.5	1.3 ± 0.1	<LOD	<LOD
Syringic acid (mg/kg DM)				
ASS	<LOD	9.1 ± 1.5	195.4 ± 49.3	191.3 ± 29.7
CIE, CAE	<LOD	<LOD	<LOD	<LOD
AJ	<LOD	<LOD	6.0 ± 0.7	15.0 ± 0.5
CIAJ	<LOD	<LOD	<LOD	14.7 ± 1.7
CAJ	<LOD	<LOD	<LOD	<LOD
Rosmarinic acid (mg/kg DM)				
ASS	<LOD	113.5 ± 63.9	<LOD	<LOD
CIE, CAE	<LOD	<LOD	<LOD	<LOD
AJ, CIAJ	<LOD	<LOD	<LOD	<LOD
CAJ	<LOD	0.6 ± 0.0	<LOD	<LOD
Trans‐cinnamic acid (mg/kg DM)				
ASS	<LOD	<LOD	<LOD	<LOD
CIE	313.8 ± 24.6	1222.2 ± 22.7	1116.1 ± 18.7	1459.9 ± 180.4
CAE	<LOD	<LOD	<LOD	<LOD
AJ, CAJ	<LOD	<LOD	<LOD	<LOD
CIAJ	291.1 ± 16.5	57.1 ± 2.3	7.7 ± 0.4	33.4 ± 4.2
Gallic acid (mg/kg DM)				
ASS, CIE	<LOD	<LOD	<LOD	<LOD
CAE	111.5 ± 4.2	1268.1 ± 42.1	437.1 ± 25.0	382.2 ± 18.7
AJ	74.0 ± 18.1	<LOD	8.5 ± 0.5	12.8 ± 1.6
CIAJ	<LOD	LOD	<LOD	<LOD
CAJ	95.1 ± 12.0	9.0 ± 1.9	6.7 ± 0.3	15.7 ± 2.1
Rutin (mg/kg DM)				
ASS, CAE	<LOD	<LOD	<LOD	<LOD
CE	0.4 ± 0.0	32.4 ± 2.4	<LOD	<LOD
AJ, CIAJ	<LOD	<LOD	<LOD	<LOD
CAJ	3.5 ± 0.2	<LOD	<LOD	<LOD
Epicatechin (mg/kg DM)				
CAE	4.43 ± 0.9	<LOD	<LOD	<LOD
ASS, CIE, AJ, CAJ, CIAJ	<LOD	<LOD	<LOD	<LOD

Abbreviations: AJ, Anjelica jam; ASS, *Angelica sylvestris* stem; CAE, carob extract; CAJ, Angelica jam fortified with carob extract; CE, cinnamon extract; CIAJ, Angelica jam fortified with cinnamon extract.

In the ASS, only caffeic acid was detected. Similarly, initial carob extract contained only trans‐cinnamic acid. On the other hand, initial carob extract has gallic acid, epicatechin, and rutin, AJ has gallic acid and caffeic acid, CIAJ has caffeic acid and trans‐cinnamic acid, and CAJ has gallic acid, caffeic acid, and rutin.

To evaluate the behavior of phenolic compounds during in vitro gastrointestinal digestion, three fractions were considered: post‐gastric fraction (PG), dialyzed fraction (Inside 1the membrane, IN), and non‐dialyzed fraction (OUT). Some of the detected phenolic compounds appeared in post‐gastric fraction or only in the intestinal phases. While syringic acid appeared in the post‐gastric phase of raw material, in the intestinal IN and OUT phases of AJ and intestinal OUT phase of CIAJ, rosmarinic acid was only detected in the post‐gastric phases of raw material and CAJ.

Increasing gallic acid and trans‐cinnamic acid concentrations were determined in post‐gastric fractions of CAE and CIE, respectively. The increasing trend of some phenolic compounds after exposure to high acidic conditions in the gastric step can be explained as the release of polyphenols linked to other compounds in the plant matrix. Transformations promoted by gastric conditions should be taken into account, because phenolics can be converted into various structural forms that can be identified or not identified (de Morais et al., 2020).

Bioaccessibility of the gallic acid from the intestinal barrier in CAE was 392%, while in CAJ, it was 7%. Similarly, bioaccessibilities of trans‐cinnamic acid after in vitro intestinal digestion were 355.6% and 2.6% in CIE and CIAJ, respectively. On the other hand, bioaccessibility of caffeic acid was 41.4% in ASS; however, it was not detected in any other IN fraction of Angelica jams. Our findings after intestinal digestion are in accordance with those of several studies (Bermúdez‐Soto et al., 2007; Celep et al., 2015; Gayoso et al., 2016; Kamiloglu et al., 2015; Siracusa et al., 2011). The high losses in dialyzed fraction could be associated with the neutral pH value, bile salts, and instability of polyphenols in aqueous solution. Most of the phenolic compounds are degraded or transformed into many other compounds after intestinal digestion due to the slightly alkaline conditions in the small intestine (Bermúdez‐Soto et al., 2007; Gayoso et al., 2016; Vallejo et al., 2004). Furthermore, high sugar content in jam samples might also affect the diffusion of polyphenols through the intestinal mucosa. High sugar levels in the samples allow the diffusion of aqua from the dialysis membrane to the food phase and this causes decrease in the volume of the dialyzed fraction (Kamiloglu et al., 2015).

## 4 CONCLUSION

Angelica jam is a forgotten traditional product from Bursa that may attract consumers' attention due to the potential antioxidant and mineral content of *A. sylvestris* L. Today, sustainable agriculture is a very important concept in terms of creating a society that meets the needs of future generations without endangering them. In order to increase the market share of traditional food products and ensure sustainability, it is necessary to make traditional food products safe, healthy, or beneficial through different innovations and to increase the recognition of forgotten traditional tastes.

To the best of our knowledge, current research represents the first study in the literature to evaluate the descriptive physicochemical and bioactive properties of Anjelica jam fortified with cinnamon and carob extracts. Although cinnamon and carob extracts were added into the formulation at low percentage, it was observed that they increased the bioactive properties in jam significantly. Therefore, future studies can include the investigation of different types of aromatic and medical plant extracts that can be incorporated into traditional jam formulations to enhance their functional properties and increase product diversity.

Furthermore, future studies should focus on optimizing traditional jam formulations and storage conditions to meet commercial production regulations.

## AUTHOR CONTRIBUTIONS

**Elif Koç Alibaşoğlu:** Conceptualization (equal); data curation (equal); formal analysis (equal); investigation (equal); validation (equal); writing – original draft (equal). **Büşra Acoğlu Çelik:** Conceptualization (equal); data curation (equal); formal analysis (equal); investigation (equal); validation (equal). **Fatma Duygu Ceylan:** Conceptualization (equal); data curation (equal); formal analysis (equal); investigation (equal); supervision (equal); validation (equal); writing – original draft (equal). **Özüm Özoğlu:** Conceptualization (equal); data curation (equal); formal analysis (equal); investigation (equal); supervision (equal); validation (equal); writing – original draft (equal). **Ertürk Bekar:** Conceptualization (equal); data curation (equal); formal analysis (equal); investigation (equal); supervision (equal); validation (equal). **Esra Çapanoğlu:** Methodology (equal); project administration (equal); writing – review and editing (equal). **Canan Ece Tamer:** Methodology (equal); project administration (equal); writing – review and editing (equal). **Mihriban Korukluoğlu:** Methodology (equal); project administration (equal); writing – review and editing (equal). **Ömer Utku Çopur:** Methodology (equal); project administration (equal); writing – review and editing (equal). **Perihan Yolci Ömeroğlu:** Funding acquisition (equal); methodology (equal); project administration (equal); validation (equal); visualization (equal); writing – original draft (equal); writing – review and editing (equal).

## FUNDING INFORMATION

This research was supported by the Scientific Research Project Office of Bursa Uludag University, Bursa, Turkey (HDP (BTUAM)‐2019/2).

## CONFLICT OF INTEREST STATEMENT

The authors have no competing interests to declare that are relevant to the content of this article.

## Data Availability

Data available on request from the authors.
